# Development, characterization, and first application of a resonant laser secondary neutral mass spectrometry setup for the research of plutonium in the context of long-term nuclear waste storage

**DOI:** 10.1007/s00216-021-03350-3

**Published:** 2021-05-10

**Authors:** Daniela Schönenbach, Felix Berg, Markus Breckheimer, Daniel Hagenlocher, Pascal Schönberg, Raphael Haas, Samer Amayri, Tobias Reich

**Affiliations:** 1 Department of Chemistry, Johannes Gutenberg-Universität Mainz, 55099 Mainz, Germany; 2 Helmholtz-Institut Mainz, 55099 Mainz, Germany; 3 GSI Helmholtzzentrum für Schwerionenforschung GmbH, 64291 Darmstadt, Germany

**Keywords:** Plutonium, Laser-SNMS, Resonant laser ionization, Ultra-trace analysis, TOF-SIMS, Nuclear waste

## Abstract

Plutonium is a major contributor to the radiotoxicity in a long-term nuclear waste repository; therefore, many studies have focused on interactions of plutonium with the technical, geotechnical, and geological barriers of a possible nuclear waste storage site. In order to gain new insights into the sorption on surfaces and diffusion of actinides through these complex heterogeneous materials, a highly sensitive method with spatial resolution is required. Resonant laser secondary neutral mass spectrometry (Laser-SNMS) uses the spatial resolution available in time-of-flight secondary ion mass spectrometry (TOF-SIMS) in combination with the high selectivity, sensitivity, and low background noise of resonance ionization mass spectrometry (RIMS) and is, therefore, a promising method for the study and analysis of the geochemical behavior of plutonium in long-term nuclear waste storage. The authors present an approach with a combined setup consisting of a commercial TOF-SIMS instrument and a Ti:sapphire (Ti:Sa) laser system, as well as its optimization, characterization, and improvements compared to the original proof of concept by Erdmann et al. (2009). As a first application, the spatial distributions of plutonium and other elements on the surface of a pyrite particle and a cement thin section were measured by Laser-SNMS and TOF-SIMS, respectively. These results exemplify the potential of these techniques for the surface analysis of heterogeneous materials in the context of nuclear safety research.

**Supplementary Information:**

The online version contains supplementary material available at 10.1007/s00216-021-03350-3.

## Introduction

Due to the long half-lives of its isotopes, such as ^239^Pu (2.41 × 10^4^ a) and ^242^Pu (3.75 × 10^5^ a), plutonium is considered one of the major contributors to the long-term radiotoxicity of nuclear waste. Its complex aqueous chemistry makes the study of its geochemical interactions with technical and geotechnical barriers, as well as the host rock system, vital for the safety assessment of possible sites for long-term storage of nuclear waste [1]. The current plans for a future German nuclear waste repository involve a multi-barrier concept in a deep geological formation storing high-level nuclear waste for one million years, with the possibility of retrieval for 500 years. Among others, clay rock is considered as a possible host rock, and cementitious materials will be part of the technical barrier [2]. Migration and sorption studies evaluating clay rock and cementitious materials as geological and technical barriers for long-term nuclear waste storage are conducted using environmentally relevant actinide concentrations (10^−7^ to 10^−9^ mol/L) [3–6]. Investigating the interactions of radionuclides with these exceedingly heterogeneous materials requires an analytical method with both high spatial resolution and sensitivity. Due to the long half-lives of the aforementioned plutonium isotopes, common radiometric analytical tools, such as alpha-spectroscopy, do not provide sufficient sensitivity. Several mass spectrometric methods adequate for a limit of detection (LOD) of 10^5^ to 10^8^ atoms, such as thermal ionization mass spectrometry (TIMS) [7] or inductively coupled plasma mass spectrometry (ICP-MS) [8], suffer from isobaric interferences. While accelerator mass spectrometry (AMS) and resonance ionization mass spectrometry (RIMS) [9, 10] are able to address this problem and provide the necessary sensitivities, they require extensive sample preparation and are only used for bulk analysis without any spatial information. Secondary ion mass spectrometry (SIMS) is a spatially resolved method, but it can be hampered by isobaric interferences and matrix effects [11].

The analytical method described here aims to combine the spatial resolution of SIMS with the highly selective resonant photoionization applied in RIMS. RIMS requires the analyte in an atomic state. During the sputtering process in SIMS, the vast majority of the eroded surface material is not ionized and, therefore, lost for detection. Different approaches to make these so-called secondary neutrals (SNs) available for mass spectrometry via post-ionization and to overcome the disadvantages of SIMS were developed in the past. Electron beam and plasma secondary neutral mass spectrometry (SNMS) [12, 13] both make use of ionization through electron impact. Electron beam SNMS has been shown to provide useful SN yields around 10^−8^ [14]. For plasma SNMS, a useful yield of around 10^−5^ has been reported [15, 16]. Furthermore, several attempts using non-resonant laser ionization of SNs have been made, which resulted in useful yields of 1% [17] or more [18, 19]. Nevertheless, these methods also suffer from isobaric interferences. Resonant photoionization suffers from a loss in analytical flexibility since it is element and even isotope specific, but due to higher cross sections it results in even higher useful yields compared to the non-resonant process [15]. Resonant Laser-SNMS has already been successfully applied to various sample types such as crystalline materials, doped semi-conductors, ocean sediments, and organic polymers [20–25], and has proven to be adequate for the analysis of radioactive samples [26–30] using fully custom-built TOF-MS as well as adapted commercially available TOF-SIMS instruments in combination with a wide range of laser systems.

For the initial tuning of the system for conducting and non-conducting sample types, samples with electrodeposited ^239^Pu on titanium foil and ^239^Pu(IV) solution dropped onto ceramic platelets were analyzed. In addition, the following two sorption samples were investigated as proof of concept for the application of Laser-SNMS in the context of nuclear safety research: (i) pyrite particles with a challenging topography were extracted from Opalinus Clay rock (OPA) and exposed to ^239^Pu(VI) solution and (ii) a thin section of hardened cement paste (HCP) was exposed to ^242^Pu(III) solution. Surface mappings obtained for both systems by TOF-SIMS and Laser-SNMS show the potential of both methods in the context of nuclear safety research.

## Experimental section

### Experimental setup

**Fig. 1:**
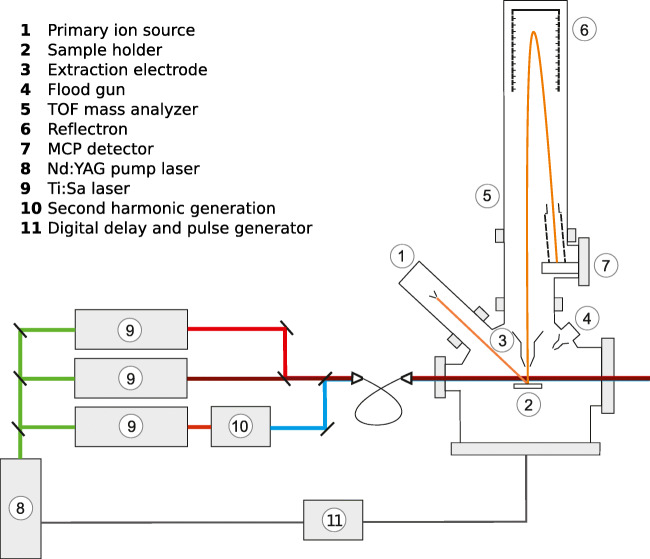
Schematic illustration of the laser secondary neutral mass spectrometry (Laser-SNMS) setup: laser system and time-of-flight secondary ion mass spectrometer (TOF-SIMS) III

The Laser-SNMS setup (Fig. 1) consisted of a commercial time-of-flight secondary ion mass spectrometer (TOF-SIMS III, IONTOF GmbH, Münster, Germany) and a custom-built Ti:sapphire (Ti:Sa) laser system. The TOF-SIMS instrument is equipped with a 25 keV ^69^Ga liquid metal ion gun (LMIG) as the primary ion source, a reflectron TOF mass analyzer, and an electron flood gun for charge compensation. The instrument control was upgraded to the IONTOF TOF-SIMS V level, including a USB-TDC and IONTOF SurfaceLab 6.6 instrument control and analysis software.

The laser system consisted of three tunable Ti:Sa lasers jointly pumped by a single frequency-doubled Nd:YAG laser (Photonics Industries International Inc., Gröbenzell, Germany, model DM532-60) at 532 nm, with 45 W of total output power at 10 kHz. The TOF-SIMS instrument and the laser system were synchronized by feeding the TOF-SIMS master timing signal into a pulse generator (BNC, San Rafael, USA, model 577). This device generated the trigger signal for the laser system which allowed for a variable delay of the laser pulses with reference to the TOF-SIMS duty cycle. Laser wavelengths were monitored using an LM007 Wavemeter (ATOS). The laser system was described in greater detail in previous publications [31, 32].

**Fig. 2:**
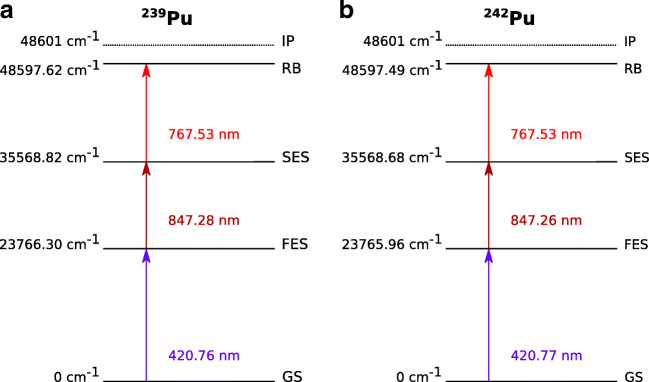
Excitation schemes for the three-step resonant ionization of ^239^Pu (**a**) and ^242^Pu (**b**) [33] from the ground state (GS) using a first and second excitation step (FES and SES) and a Rydberg state (RB) just below the ionization potential (IP)

The excitation schemes for resonant ionization used in this work are presented in Fig. 2 [33]. The laser light was transmitted to the TOF-SIMS instrument via an optical fiber and introduced into the photon-SN interaction region through a CF viewport. A telescope on a 5-axis translation and rotation stage was used for focusing and positioning the beam directly under the extraction electrode of the TOF-SIMS. The use of an optical fiber allowed for reproducible beam positioning, but limited the minimum focus diameter to approximately 1 mm due to the beam divergence at the end of the fiber. During standard SIMS operation, the distance between the sample surface and the extraction electrode is 1.5 mm. This distance was increased to 2.5 mm for both TOF-SIMS and Laser-SNMS in order to prevent the diverging laser beam from hitting the sample and sample holder. In addition, a newly designed pedestal sample holder allowed for samples to be positioned and moved safely under the laser interaction volume.

### Samples and preparation

Different samples were prepared and analyzed with TOF-SIMS and Laser-SNMS to account for the various types of materials encountered in the context of a long-term nuclear waste repository. This approach considers influences from conducting and non-conducting surfaces, as well as sample topography. The respective preparation methods are described below. All samples were attached to the pedestal sample holder using conducting double-sided adhesive carbon tape.

### Conducting samples

#### Synthetic, electrodeposited sample

For the purpose of adapting and optimizing the measurement parameters of the TOF-SIMS instrument for Laser-SNMS on the conducting samples, ^239^Pu was electrodeposited as a 5-mm diameter spot onto Ti filaments. For the electrolysis, 0.2 g/mL of aqueous (NH_4_)_2_SO_4_ electrolyte solution was used, and a current of ~250 mA at 17 V was applied for 1.5 h. Under these conditions, Pu is deposited as a hydroxide. An efficiency for the electrolysis of around 86% was determined via alpha-spectroscopy (partially depleted silicon detector, CR-SNA-450-100, 450 mm^2^, 20 keV resolution at 5.486 MeV, Ortec, Germany) for a sample prepared with 10^11^ atoms of ^239^Pu in the electrolyte solution. Assuming a homogeneous distribution of the Pu layer, the surface loading of a sample can be estimated. The custom electrolysis setup is described in detail in previous publications [32, 34].

#### Semi-synthetic, topographic samples

The Fe(II)-bearing mineral pyrite has been identified in previous sorption experiments as a redox-active component of Opalinus Clay rock (OPA), that is involved in the reduction and immobilization of Pu [3, 35]. Micrometer-sized pyrite particles were extracted from calcite-saturated, aqueous suspensions of OPA and manually sorted under a microscope. The particles were then contacted for 4 days with 1 mL of a ^239^Pu(VI) solution (2 × 10^−5^ mol/L, pH 7.8) in OPA pore water [36] saturated with calcite as a background electrolyte. All experiments with wet chemistry were conducted under an argon atmosphere. Afterwards, the particles were extracted from the solution and left to dry on filter paper and their morphology and size characterized via scanning electron microscopy (SEM, Philips XL 30). For TOF-SIMS and Laser-SNMS measurements, the primary ion (PI) beam was rastered in sawtooth movement over an area of 400 × 400 μm^2^ which included the particle at a resolution of 512 × 512 px and a PI current of 2 μA (LMIG) at a pulse width of 6.5 ns (TOF-SIMS) and 90 ns (Laser-SNMS) with 1 shot/px. The extraction delay was set to 1.165 μs for SIMS and 1.650 μs for SNMS mode with a total duty cycle of 100 μs.

### Non-conducting samples

#### Synthetic, ceramic samples

In order to account for measurements on non-conducting materials encountered in the context of a nuclear waste repository, e.g., clay rock and cementitious materials, specific operational parameters were determined for insulating surfaces. Two microliters of a ^239^Pu solution (6 × 10^−4^ mol/L in 6 mol/L HNO_3_) were applied in 20 nL droplets on smooth glass–ceramic discs (Macor®, ⌀ = 7 mm, 1 mm thickness) using a drop-on-demand inkjet printing system [37].

#### Semi-synthetic, non-topographic sample

An HCP sorption sample was produced using Portland cement (OPC, CEM I, Dyckerhoff GmbH, Wiesbaden, Germany), prepared according to DIN EN 196-3 and hardened in water for at least 28 days. A thin section (9 mm × 7.5 mm) was prepared, embedded in epoxy resin (EpoxiCure 2 Resin and EpoxiCure 2 Hardener, Buehler, Lake Bluff, IL, USA), and attached to a glass carrier plate (⌀ = 10 mm). The surface of the thin section was conditioned in a custom sorption cell with artificial cement pore water (ACW) [38] for 24 h. Afterwards, it was contacted with 5 mL of ^242^Pu(III)-ACW solution (2.13 × 10^−5^ mol/L, pH 13) for 72 h under an argon atmosphere. This concentration of Pu was well above the solubility limit, but additional experiments with the sample required an excess of the analyte. After drying, around 10^15^ atoms of ^242^Pu were detected on the surface of the thin section using alpha-spectroscopy. TOF-SIMS and Laser-SNMS measurements were conducted using the electron flood gun for charge compensation and applying a surface potential of −140 V. The HCP sample was rastered in random mode with a field-of-view of 500 × 500 μm^2^ at a resolution 256 × 256 px with 10 shots/px at 2 μA PI current (LMIG) and a pulse width of 13.5 ns for TOF-SIMS and 150 ns for Laser-SNMS at 10 kHz repetition rate with no extraction delay in case of TOF-SIMS and 1.550 μs for Laser-SNMS.

## Results and discussion

### Characterization of the Laser-SNMS setup

#### Settings for conductive and non-conductive samples

The initial adaptation of the system for Laser-SNMS, as well as the laser beam and laser focus positioning and adjustment of timing to retrieve a first resonant signal, was carried out using a Gd foil, and the resonant excitation scheme for Gd described in [27]. After a first resonant signal had been achieved, the operational parameters were optimized for Pu.

**Fig. 3:**
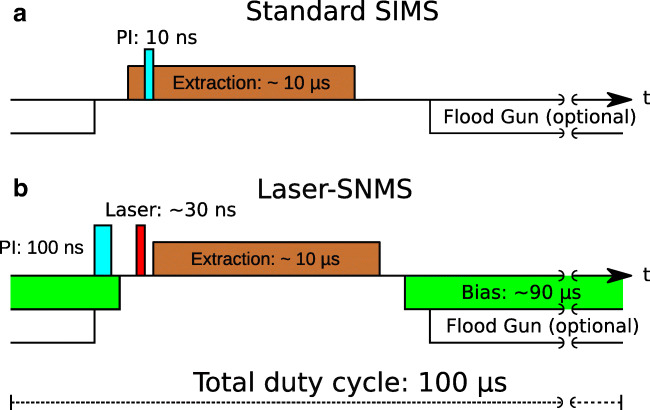
Timing schemes for non-delayed SIMS (**a**) and Laser-SNMS (**b**) (not to scale)

Changes to the operational parameters of the instrument included the LMIG ^69^Ga PI beam focus and position, as well as the mass analyzer to account for the increased distance of 2.5 mm between the sample surface and the extraction electrode and the different points of origin for secondary and laser ions. The PI pulse width for Laser-SNMS measurements was increased compared to 10 ns in TOF-SIMS mode in order to produce more SNs in the laser interaction volume, which resulted in an increase in photo ions, as displayed in Fig. S1 in the Supplementary information (ESM). The timing sequence of primary ion pulse, laser pulse, and laser ion extraction had to be set up for Laser-SNMS operation. Timing schemes for SIMS and Laser-SNMS are displayed in Fig. 3.

In standard SIMS mode, the extraction voltage is applied to the extraction electrode before the PIs hit the sample surface. All subsequently emitted secondary ions (SIs) are immediately accelerated into the mass analyzer, which results in high transmission and increased mass resolution. The extraction voltage is applied for approximately 10 μs. The application of the extraction voltage can be delayed via the SurfaceLab software. In SIMS mode, this is used to delay the extraction of the secondary ions until they have drifted further away from the sample surface. This compensates the distortion of the equipotential lines of the extraction field due to sample topography and the resulting loss of SIs. The principle of delayed extraction was repurposed here for Laser-SNMS operation. In Laser-SNMS mode, the extraction of laser ions is delayed while a repelling voltage (extraction bias) is applied to the extraction electrode, deflecting SIs from the mass analyzer’s acceptance volume while the SNs drift freely into the laser interaction volume. Afterwards, the approximately 30 ns laser pulse is applied after disengaging the bias voltage. The SNs are resonantly photoionized and accelerated into the mass analyzer. The fine-tuning of the laser timings in relation to the extraction of ions is crucial, as displayed by the changes in the ^239^Pu signal intensity for a conducting sample in Fig. S2 (see ESM). In case of our setup, a laser pulse within a few nanoseconds of the extraction pulse resulted in the highest signal intensity.

The development of new measurement settings for Laser-SNMS described above was carried out for both the electrodeposited ^239^Pu on Ti foil for conducting samples, and the glass-ceramic discs for non-conducting samples, which were then used for the analysis of the respective semi-synthetic model system. In addition to adapting the operational parameters of the mass analyzer for insulating surfaces, electron flooding using an electron flood gun was deployed for charge compensation, and the raster mode of the LMIG was changed from a sawtooth to a random pattern in order to prevent further local charge buildup. Depending on the sample type, a delay of the laser pulse of −150 to 0 ns in reference to the ion extraction pulse of the mass analyzer was used for subsequent Laser-SNMS measurements.

#### Saturation powers

**Fig. 4:**
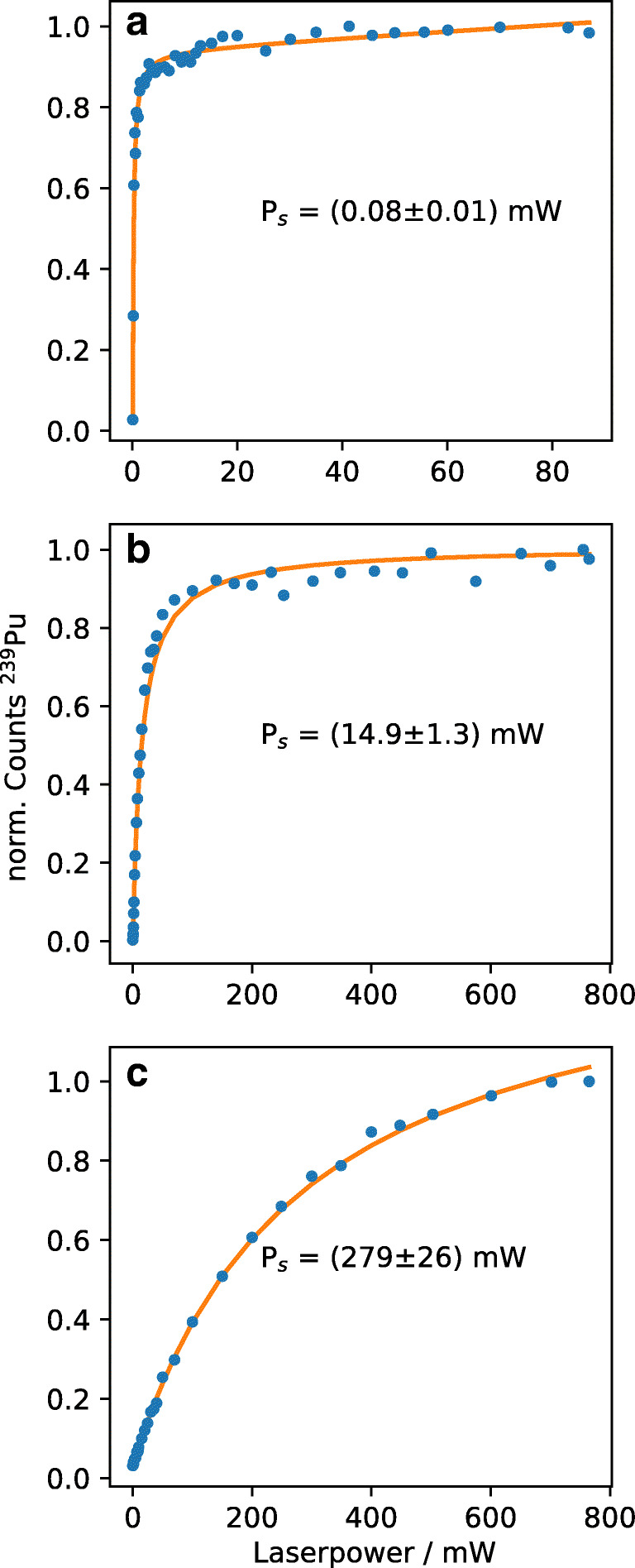
Saturation curves and fits for the first (**a**) and second (**b**) excitation steps, the ionizing step (**c**), and the respective saturation powers, P_s_

The saturation powers, P_s_, presented in Table 1 were determined using an electrolysis sample with a surface load of 5.6 × 10^9^ atoms per μm^2^ of ^239^Pu. The power of one laser was varied while the other two lasers remained at full power. Despite the third step not reaching saturation, the data were fitted using an extended saturation curve given in Eq. 1 [39]. P_s_ is the saturation power, A the maximum amplitude of the curve, and I_0_ describes the offset caused by the non-resonant ionization generated by other steps, whereas the linear term takes non-resonant ionization and broadening of the beam with increasing laser power into account. Fits and curves are presented in Fig. 4.

1 $$ \mathrm{I}={\mathrm{I}}_0+\mathrm{A}\cdotp \frac{\mathrm{P}/{\mathrm{P}}_{\mathrm{s}}}{1+\mathrm{P}/{\mathrm{P}}_{\mathrm{s}}}+\mathrm{m}\cdotp \mathrm{P} $$

**Table 1:** Saturation powers, P_s_, and applied powers, P_a_, for the three excitation steps of Pu

Step	P_s_ (mW)	P_a_ (mW)
First	0.08 ± 0.01	30
Second	14.9 ± 1.3	450
Ionizing	279 ± 26	720

The measurements in this work were performed using higher laser powers, P_a_, in order to maximize the ionization yield. Due to the monoisotopic nature of the samples, saturation broadening can be neglected. However, the first and second excitation steps were attenuated to 30 mW and 450 mW, respectively, to decrease non-resonant ionization and to improve the signal-to-noise ratio. The laser for the ionizing step was usually operated at a power of around 720 mW.

#### Efficiency of the Laser-SNMS setup

A proof of concept for Laser-SNMS on Pu was presented by Erdmann et al. with the same TOF-SIMS III instrument used in this work before the upgrade in 2009 [27]. Without rastering the PI beam over the sample surface, which ensures complete depletion of Pu in the affected area within a reasonable time, an overall efficiency of ≥10^−7^ was established by Erdmann et al. for a sample with a surface load of 3.5 × 10^7^ atoms per μm^2^ of ^242^Pu and assuming a PI beam focus diameter of 6 μm. Repeating the same experiment twice with the now upgraded setup for an electrodeposition of 7.89 × 10^3^ atoms per μm^2^ of ^239^Pu resulted in an average efficiency of (7.2 ± 0.8) × 10^−4^. In an additional measurement, the PI beam was rastered over an area of 18 × 18 μm^2^ of the same sample, and an overall efficiency of (8.8 ± 0.3) × 10^−4^ was obtained. This increase in efficiency by three orders of magnitude compared to Erdmann et al. can be attributed to the decreased distance between the extractor electrode and the sample surface (now 2.5 mm compared to 7–8 mm) and the extended optimization of the measurement parameters.

#### First application

To assess the capabilities of a combined approach of TOF-SIMS and Laser-SNMS for the analysis of Pu in the context of a long-term nuclear waste repository, two semi-synthetic model systems were chosen. Pyrite particles present a very challenging topography, but they can be measured without charge compensation, whereas the HCP thin section has a favorable flat surface but low or no conductivity. The measurements presented here serve as a proof of concept with regard to future experiments.

**Fig. 5:**
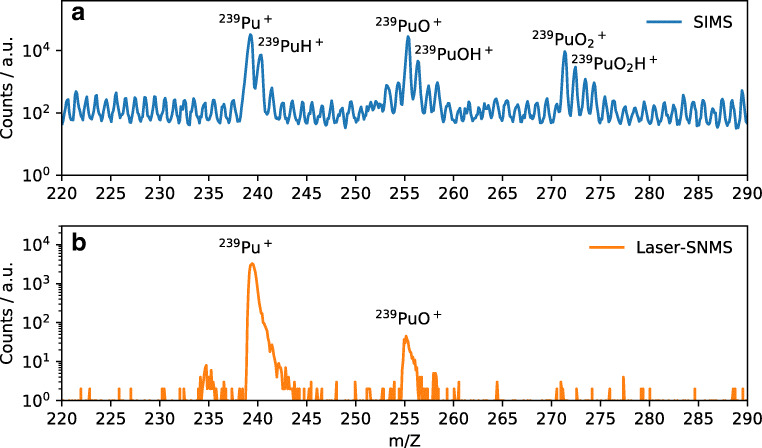
Sections of spectra obtained in SIMS mode (**a**) and Laser-SNMS mode (**b**) showing the ^239^Pu signal of a contacted pyrite particle. The SIMS spectrum shows signals for ^239^Pu, ^239^PuO, and even ^239^PuO_2_, as well as their respective hydrides, while the Laser-SNMS spectrum only features resonantly excited ^239^Pu and non-resonant ^239^PuO. Binning increment of 0.1 for both **a** and **b**

**Fig. 6:**
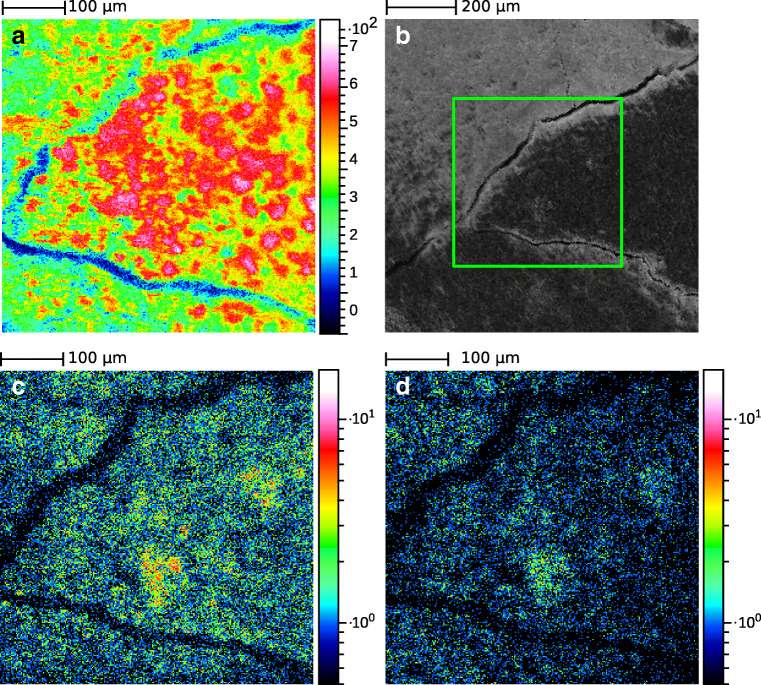
Total secondary ion image (**a**) and SEM image (**b**) of a section of the non-conducting HCP sorption sample after contact with ^242^Pu(III) solution. Spatial distribution of ^242^Pu in SIMS (**c**) and Laser-SNMS mode (**d**). The green square in **b** represents the area scanned in TOF-SIMS and Laser-SNMS

**Fig. 7:**
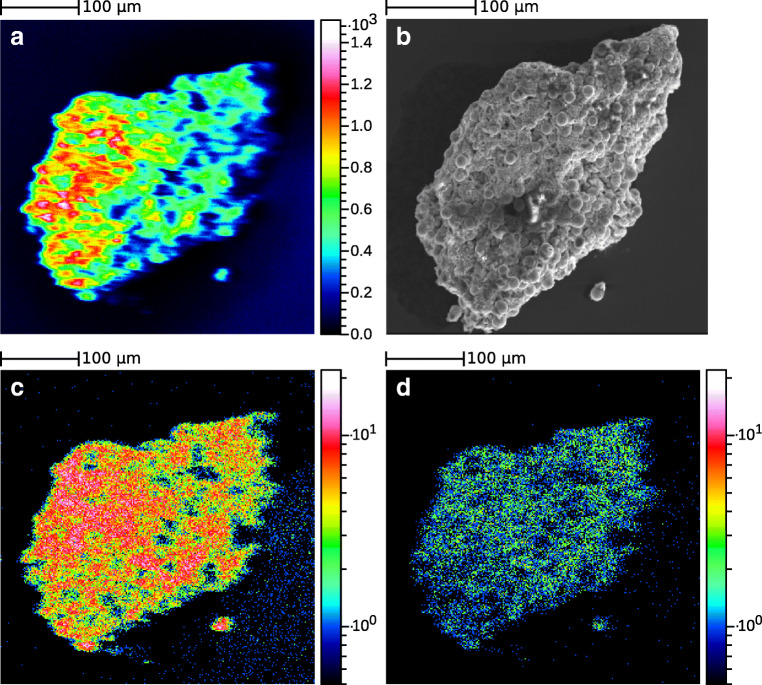
Total secondary ion image (**a**) and SEM image (**b**) of a pyrite particle extracted from Opalinus Clay rock and contacted with ^239^Pu(VI) solution. Spatial distribution of ^239^Pu in SIMS (**c**) and Laser-SNMS mode (**d**). The mass images have been binned by a factor of 4

Figure 5 shows a section of the spectrum with ^239^Pu contacted pyrite particle in SIMS mode and Laser-SNMS mode. The loss of mass resolution for Laser-SNMS compared to TOF-SIMS is due to an increased extraction delay by 485 ns and the nature of the ionization process. Whereas the SIs are created on PI impact, the photoionization of the SNs takes place in a volume above the sample. When extracted, the increased spatial distribution of the photo ions compared to the SIs results in peak broadening. The strength of Laser-SNMS lies in the reduction of background and, therefore, the increased signal-to-noise ratio by a factor of 15 for this measurement. Detuning the first excitation step allows for background measurements as well as confirmation that the signal observed is indeed the analyte. But this high selectivity also comes with a disadvantage: While with TOF-SIMS it is possible to detect ^239^Pu^+^, ^239^PuO^+^, and ^239^PuO_2_^+^ as well as their respective hydrides, this analytical flexibility is lost for Laser-SNMS due to the nature of the ionization process that requires ^239^Pu^0^. Here, only ^239^Pu^+^ and non-resonantly ionized ^239^PuO^+^ are observed. In fact, while TOF-SIMS can detect all components on the sample surface, resonant Laser-SNMS is limited to a single analyte without changing the excitation wavelengths. Furthermore, as Pu is expected to be present as hydroxo species, the majority of the analyte might be sputtered as PuO or PuO_2_ and, therefore, not detected using photoionization, as has been demonstrated for uranium [40]. This might also play a role when looking at the differences in the mass images obtained for Pu via TOF-SIMS and Laser-SNMS for the two semi-synthetic model systems. Figures 6 and 7 display the lateral distributions of Pu obtained from TOF-SIMS and Laser-SNMS measurements as well as the total ion distribution in SIMS mode and the SEM images of an area of the HCP thin section and a pyrite particle, respectively. For both samples, SIMS and Laser-SNMS showed a similar distribution of Pu on the sample surface. Areas of increased signal intensity can be identified in both SIMS and Laser-SNMS images, but a significant loss in signal intensity is observed for Laser-SNMS compared to SIMS which can be explained by the aforementioned lack of secondary neutral Pu for photoionization. This results in longer data acquisition times or an insufficient signal for the analysis of the lateral distribution.

Additional differences observed in the mappings might be caused by isobaric interferences present in the SIMS measurement, as well as the influence of the chemical environment on the sputter yield, the so-called matrix effect, which was hoped to be reduced for Laser-SNMS [41], but studies have shown that it cannot be eliminated [40, 42, 43]. While no influence of topography was observed in TOF-SIMS mass mappings for the flat HCP thin section (Fig. 6 and ESM Fig. S3), in case of the pyrite particle (Fig. 7 and ESM Fig. S4), the signal might also be disturbed by the strong topography, which is a known challenge for TOF-SIMS [11, 44], and it has also been observed for Laser-SNMS [45]. Local extraction field distortions caused by a non-flat surface hinder homogenous ion extraction from the observed area. The TOF-SIMS mappings for a selection of elements of the pyrite particle presented in ESM Fig. S4 display such a dependency of the ion signal on the geometry of the sample. Nevertheless, since sputtered neutrals get photoionized in a volume above the sample and not on its surface, the effect should be greatly reduced in Laser-SNMS mode.

To evaluate geochemical interactions between Pu and materials encountered in a nuclear waste repository in future experiments, we propose a combined approach using the analytical flexibility of TOF-SIMS to identify components of the material’s surface and the high sensitivity and selectivity of Laser-SNMS. Despite all challenges, Laser-SNMS allows for highly selective detection of Pu on the sample surface and, therefore, enhances the analytical capabilities of TOF-SIMS.

Despite that no correlation between surface materials and Pu in both semi-synthetic samples presented in this study has been observed, possibly due to the still relatively high concentrations of Pu used in the sample preparations, the combination of TOF-SIMS and Laser-SNMS offers a new and promising tool for nuclear safety research. Both methods do not require a large facility and can be used without extensive sample preparation in rapid succession by simply loading different operational parameters for the instrument. In addition, the specimen is still accessible for analysis by other means. Despite the material surface being slowly damaged by the PI beam during the measurement, this is limited to the field of view and can be reduced to a minimum.

## Conclusion

In this work, a system for resonant Laser-SNMS based on a commercial TOF-SIMS III instrument combined with three tunable Ti:Sa lasers for resonant photoionization was presented. Measurement settings were developed for the analysis of both conducting and non-conducting samples in contact with Pu, and significant improvements in the overall efficiency of the setup were made compared to the original proof of concept. Laser-SNMS was used for the analysis of model systems aimed to gain insight into the complex geochemistry expected in a long-term nuclear waste repository. The resonant Laser-SNMS allowed for significant background suppression and an improved signal-to-noise ratio, improving the identification of Pu even in the presence of isobaric interferences. However, challenges regarding the sputter rate of atomic Pu from the presented samples were identified and require further study. Future studies might include the analysis of diffusion samples, retrieving the diffusion parameters necessary for modeling migration processes in a long-term nuclear waste repository, or the simultaneous measurement of different radionuclides present in one sample. Since multi-step resonant excitation schemes are already available for other elements and isotopes, the method can easily be transferred to other radionuclides, such as Np [39], Tc [32], or U [46], contributing to the radiotoxicity present in a nuclear waste repository. In conclusion, the combined approach of TOF-SIMS and resonant Laser-SNMS offers a promising tool for nuclear safety research.

## Supplementary Information

ESM 1 (PDF 2912 kb)

## Data Availability

Raw data were generated at the Department of Chemistry, Johannes Gutenberg-Universität Mainz, 55099 Mainz, Germany. Derived data supporting the findings of this study are available from the corresponding author, T.R., upon request.
