# Supplementary material for: Development, characterization, and first application of a resonant laser secondary neutral mass spectrometry setup for the research of plutonium in the context of long-term nuclear waste storage
Source: Anal Bioanal Chem. 2021 May 10;413(15):3987–97. doi: 10.1007/s00216-021-03350-3 (PMC8189947; doi:10.1007/s00216-021-03350-3)
Supplement: Supplementary file 1 — (PDF 2912 kb) [file 216_2021_3350_MOESM1_ESM.pdf]

Analytical and Bioanalytical Chemistry

Supplementary Information

Development, characterization, and first application of a resonant laser secondary neutral mass spectrometry setup for the research of plutonium in the context of long-term nuclear waste storage

Daniela Schönenbach[1], Felix Berg[1], Markus Breckheimer[1], Daniel Hagenlocher[1], Pascal Schönberg[1], Raphael Haas[1,2,3], Samer Amayri[1], and Tobias Reich[1,*]

[1]Department of Chemistry, Johannes Gutenberg-Universität Mainz, 55099 Mainz, Germany

[2]Helmholtz-Institut Mainz, 55099 Mainz, Germany

[3]GSI Helmholtzzentrum für Schwerionenforschung GmbH, 64291 Darmstadt, Germany

[*]Corresponding author: Tobias Reich, Email: tobias.reich@uni-mainz.de

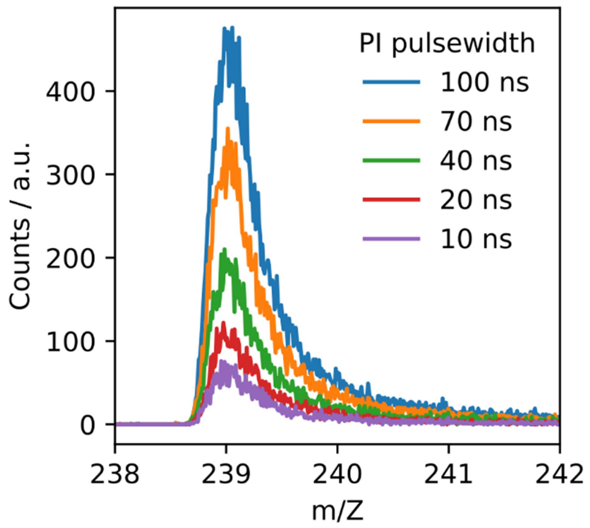

Fig. S1 Laser-SNMS signal of electrodeposited ^{239}Pu on Ti foil for different primary ion (PI) pulse widths. Binning increment: 0.01

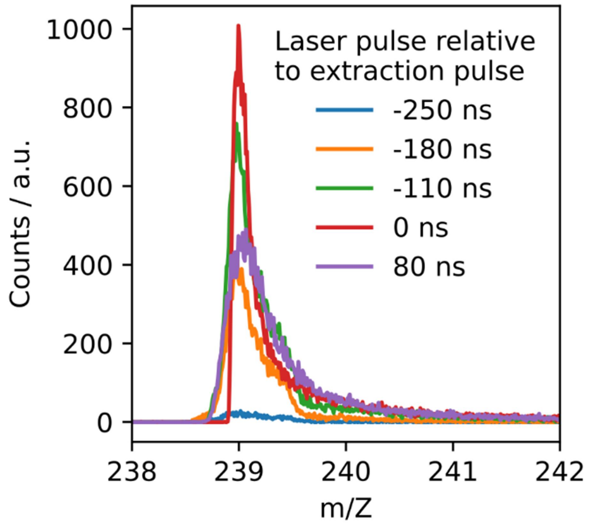

Fig. S2 Laser-SNMS signal of electrodeposited ^{239}Pu on Ti foil for different laser delay timings relative to the start of the ion extraction, as observed via an external pick-up. Negative values indicate a laser pulse before the extraction. Binning increment: 0.01

Table S1 Operational parameters for the liquid metal ion gun (LMIG) for both secondary ion mass spectrometry and resonant laser secondary neutral mass spectrometry at 2.5 mm distance between extractor and sample

Parameter	LMIG
Emission Current / µA	2
Energy / V	25000
Extractor / V	9000
Lens Source / V	3293
Lens Target / %	68.68
X Crossover / %	-17.3
Y Crossover / %	7.1
X Source / %	9.5
Y Source / %	-12.8

Table S2 Operational parameters for resonant laser secondary neutral mass spectrometry and secondary ion mass spectrometry on conducting and non-conducting surfaces at 2.5 mm distance between extractor and sample

Parameter	Laser-SNMS Conducting sample	Laser-SNMS Non-conducting sample	SIMS
Extraction delay / ns	1650	1550	0/1165
Bias / V	+500	–40/–50	0
Analyzer energy / V	1000	1900	2000
Analyzer acceleration / V	9500	9900	9500
PI pulse width / ns	90	150	6.5-10
Laser timing / ns	-150 to 0	–85	-
Raster mode	Sawtooth	Random	Sawtooth/Random
Flood gun	No	Yes	Depending on sample

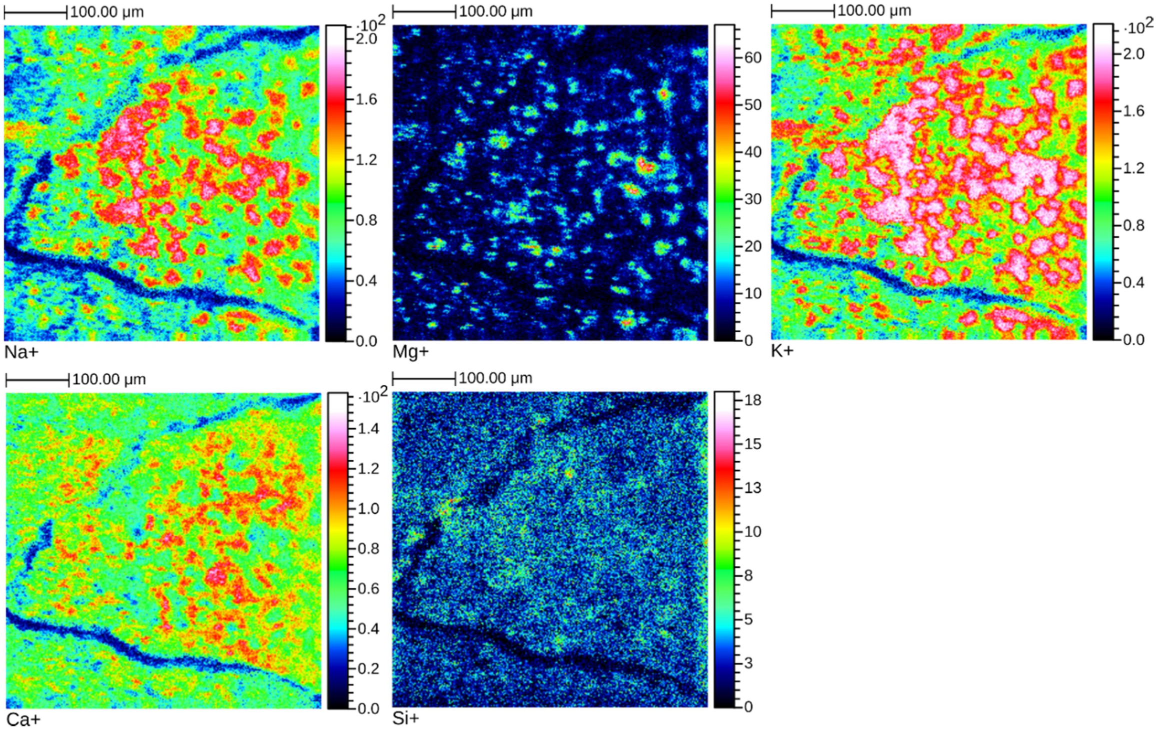

Fig. S3 TOF-SIMS mass images for selected elements of the analyzed hardened cement paste thin section

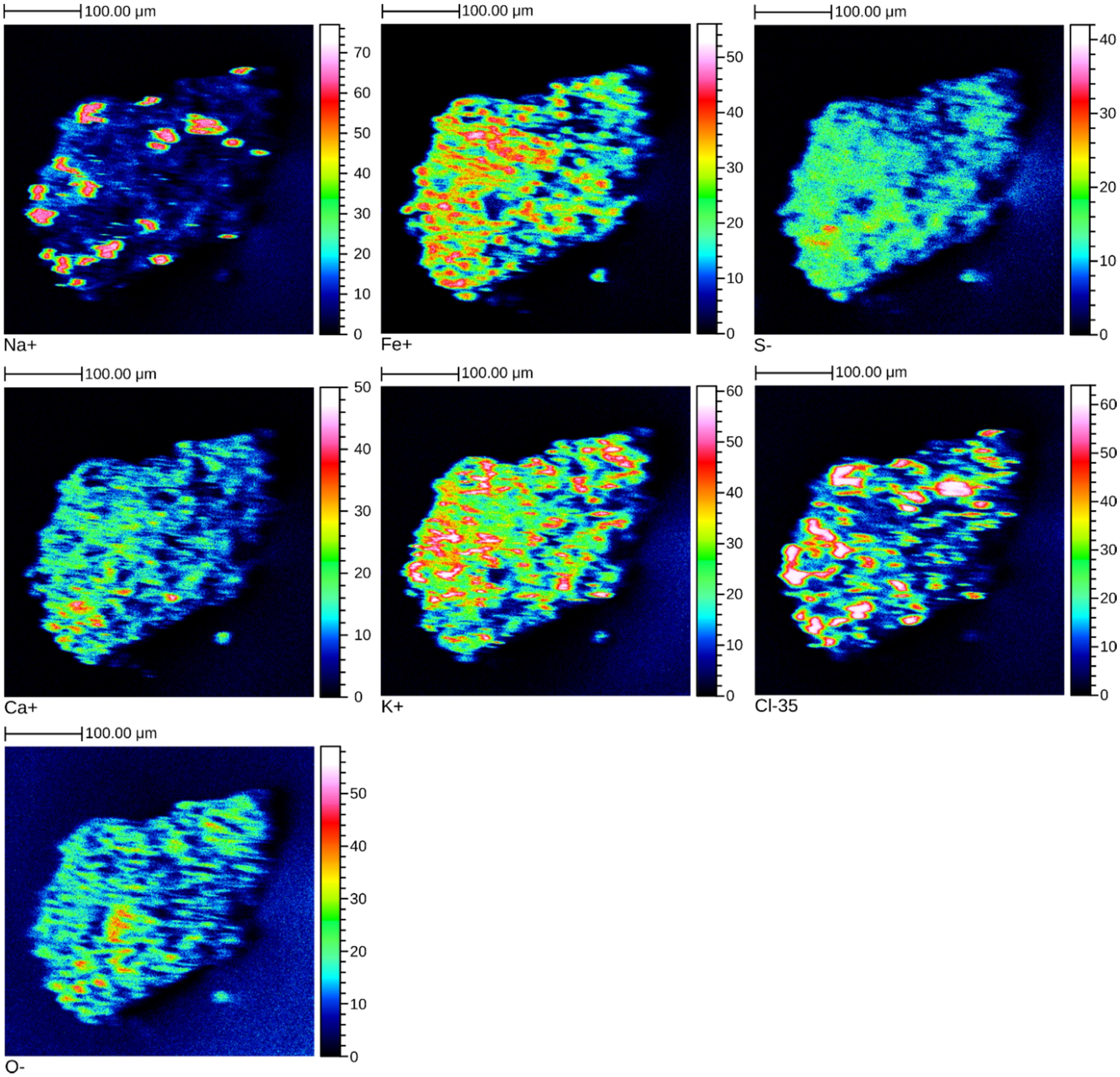

Fig. S4 TOF-SIMS mass images for selected elements of the analyzed pyrite particle
